# Implementation of treatment escalation plans in a community psychiatric hospital

**DOI:** 10.1192/bjo.2021.208

**Published:** 2021-06-18

**Authors:** Anne Yan Ting Chua, Adnaan Ghanchi, Jessica Grayston, Nida Yasmeen, Sean Insigne, Stephen Woolford, Sahan Wijayaweera, Harnish P Patel, Jay Amin, Sangeeta Makh

**Affiliations:** 1 University of Southampton; 2 Southern Health NHS Foundation Trust

## Abstract

**Aims:**

Treatment Escalation Plans (TEP) detail appropriate ceilings of care and guide treatment of patients based on shared decision making. Whilst established in many acute trusts, TEP are not frequently used in community mental health hospitals. This is particularly concerning in organic mental health wards, where patients with severe dementia may be transferred to acute hospitals for treatment without consideration about whether this is appropriate. Our aim for this quality improvement project was to develop and implement TEP within a community mental health hospital to support the management of our older patients with severe mental illness.

**Method:**

We designed a TEP form based on a prototype used in a partner acute trust and evaluated its use on our wards, comprising 20 patients between August-September 2020. This included clear options of the different ceilings of care and what they comprised of for our patients. We obtained quantitative data on the use of TEP, including the length of time from admission to completion, as well as qualitative data from healthcare staff regarding their experience of using TEP.

**Result:**

TEP implementation was feasible and well received among members of staff. All 20 patients had a TEP in place within 2 weeks of admission. The mean number of days taken to complete a TEP form in August-September was 7.1. A snapshot done 2 months later showed new admissions had a mean number of days to complete TEP reduced to 3.2. There was an improvement in understanding the purpose, comprehensiveness and location of TEP forms during their implementation. The key theme that arose from qualitative analysis of healthcare staff comments was that TEP forms provided clear guidance on the appropriateness of escalation of care.

**Conclusion:**

TEP forms offer clear guidance to treating clinicians about the ceilings of care for patients. This also allows for open conversations with patients or their next of kins regarding ceiling of care. This is especially important in mental health inpatients with dementia, when escalation of treatment is not always appropriate. TEP were successfully implemented in our community mental health hospital and we plan further post-implementation evaluation. We intend to roll out the TEP form across our mental health trust and share findings globally to promote best practice.

